# Bis[2-(thio­phen-2-yl)quinoxaline-κ*N* ^4^]silver(I) tetra­fluoridoborate

**DOI:** 10.1107/S1600536813004510

**Published:** 2013-02-23

**Authors:** Guy Crundwell

**Affiliations:** a Department of Chemistry, Central Connecticut State University, New Britain, CT 06053, USA

## Abstract

In the title compound, [Ag(C_12_H_8_N_2_S)_2_]BF_4_, the two-coordinate Ag^I^ ion lies on a crystallographic inversion center and is linearly bonded to the N-donor atoms of two separate quinoxaline ligands. The thio­phenyl ring of the ligand is nearly coplanar with the quinoxaline ring system [dihedral angle = 9.15 (13)°]. In the crystal, the complex mol­ecules pack in layers parallel to (-102) and form weak π–π ring stacking inter­actions [minimum ring centroid separation = 3.7054 (17) Å]. The tetra­fluoridoroborate anion is equally disordered about an inversion center.

## Related literature

For the synthesis of the title compound, see: Bhogala *et al.* (2003 ▶). For the structure of a similar compound, see: Wang (2012 ▶).
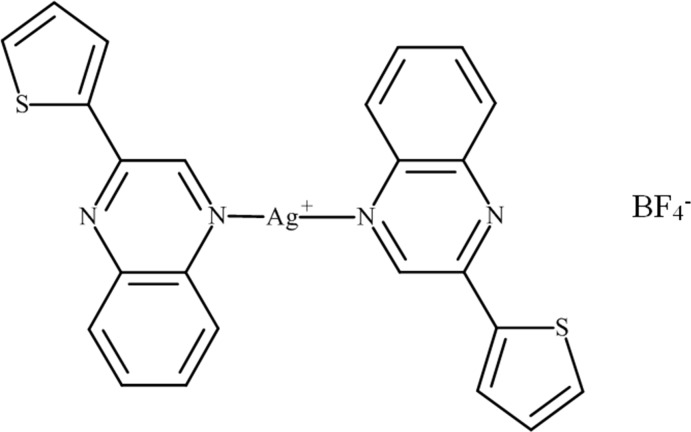


## Experimental

### 

#### Crystal data

[Ag(C_12_H_8_N_2_S)_2_]BF_4_
*M* *_r_* = 619.21
Monoclinic, 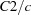

*a* = 14.1249 (19) Å
*b* = 13.1972 (16) Å
*c* = 13.739 (2) Å
β = 102.991 (15)°
*V* = 2495.5 (6) Å^3^
*Z* = 4
Mo *K*α radiation
μ = 1.03 mm^−1^
*T* = 293 K
0.32 × 0.22 × 0.17 mm

#### Data collection

Oxford Diffraction Xcalibur Sapphire3 CCD diffractometer
Absorption correction: multi-scan (*CrysAlis PRO*; Oxford Diffraction, 2009 ▶) *T*_min_ = 0.657, *T*_max_ = 1.000
30081 measured reflections
4624 independent reflections
2670 reflections with *I* > 2σ(*I*)
*R*_int_ = 0.045

#### Refinement

*R*[*F* ^2^ > 2σ(*F* ^2^)] = 0.054
*wR*(*F* ^2^) = 0.204
*S* = 0.93
4624 reflections
187 parameters
55 restraints
H-atom parameters constrained
Δρ_max_ = 0.68 e Å^−3^
Δρ_min_ = −0.51 e Å^−3^

### 

Data collection: *CrysAlis CCD* (Oxford Diffraction, 2009 ▶); cell refinement: *CrysAlis RED* (Oxford Diffraction, 2009 ▶); data reduction: *CrysAlis RED*; program(s) used to solve structure: *SHELXS97* (Sheldrick, 2008 ▶); program(s) used to refine structure: *SHELXL97* (Sheldrick, 2008 ▶); molecular graphics: *PLATON* (Spek, 2009 ▶); software used to prepare material for publication: *SHELXTL* (Sheldrick, 2008 ▶).

## Supplementary Material

Crystal structure: contains datablock(s) I, global. DOI: 10.1107/S1600536813004510/zs2248sup1.cif

Structure factors: contains datablock(s) I. DOI: 10.1107/S1600536813004510/zs2248Isup2.hkl

Additional supplementary materials:  crystallographic information; 3D view; checkCIF report

## Figures and Tables

**Fig. 1.**
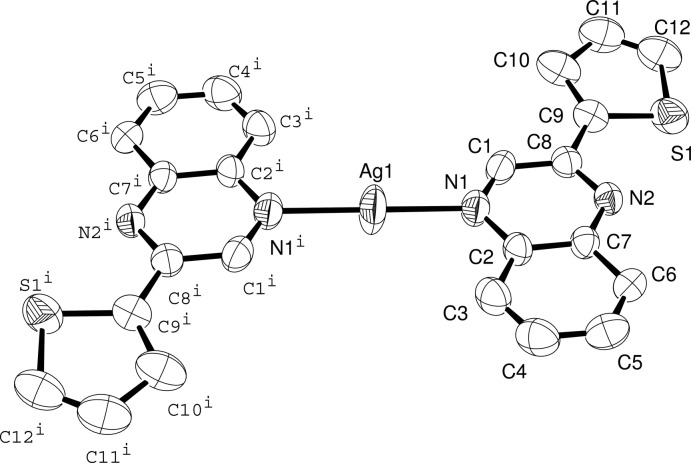
A view of the title compound (Spek, 2009). Displacement ellipsoids are drawn at the 50% probability level. The disordered tetrafluoridoroborate anion is omitted for clarity. For symmetry code (i): -x+5/2, -y+1/2, -z+1.

## supplementary crystallographic information

### Comment

In the title compound, [Ag(C_12_H_8_N_2_S)_2_] BF_4_, the Ag^+^ lies on a crystallographic inversion center and is linearly bonded to the N donor atoms of two separate quinoxaline ligands [Ag—N, 2.166 (2) Å] (Fig. 1). The thiophenyl substituent ring of the ligand is nearly coplanar with the quinoxaline ring [dihedral angle = 9.15(0.13)°]. All bond lengths and angles fall within the typical ranges found in similar complexes (Wang, 2012). In the crystal, the complex molecules pack in layers and give weak π–π ring stacking interactions [minimum ring centroid separation = 3.7054 (17) Å]. The tetrafluoroborate anion is 50% disordered about an inversion center.

### Experimental

In a 50 mL beaker, 0.471 mmol of 2-(2-thiophenyl)quinoxaline (100 mg) was added to a 20 mL of near boiling absolute ethanol. In a seperate 50 mL beaker, 0.236 mmol of AgBF_4_ (39 mg) was added to 15 mL of near boiling absolute ethanol. The two solutions were combined then pipetted into several test tubes placed inside amber vials. Over the course of days, small colourless block-shaped crystals had formed.

### Refinement

Hydrogen atoms were placed in calculated positions with a C—H distance of 0.93 Å and were included in the refinement in a riding motion approximation with *U*_iso_ = 1.2*U*_eq_ of the carrier atom. Difference maps indicated that the tetrafluoridoroborate atom was disordered about an inversion center in the lattice. The disorder was treated by the use of several similarity restraints.

### Crystal data

**Table:**

[Ag(C_12_H_8_N_2_S)_2_]BF_4_	*F*(000) = 1232
*M**_r_* = 619.21	*D*_x_ = 1.648 Mg m^−^^3^
Monoclinic, *C*2/*c*	Melting point: 395 K
Hall symbol: -C 2yc	Mo *K*α radiation, λ = 0.71073 Å
*a* = 14.1249 (19) Å	Cell parameters from 7201 reflections
*b* = 13.1972 (16) Å	θ = 4.3–33.4°
*c* = 13.739 (2) Å	µ = 1.03 mm^−^^1^
β = 102.991 (15)°	*T* = 293 K
*V* = 2495.5 (6) Å^3^	Plate, white
*Z* = 4	0.32 × 0.22 × 0.17 mm

### Data collection

**Table:**

Oxford Diffraction Xcalibur Sapphire3 CCD diffractometer	4624 independent reflections
Radiation source: fine-focus sealed tube	2670 reflections with *I* > 2σ(*I*)
Graphite monochromator	*R*_int_ = 0.045
Detector resolution: 16.1790 pixels mm^-1^	θ_max_ = 33.4°, θ_min_ = 4.3°
ω scans	*h* = −21→21
Absorption correction: multi-scan (*CrysAlis PRO*; Oxford Diffraction, 2009)	*k* = −20→20
*T*_min_ = 0.657, *T*_max_ = 1.000	*l* = −21→20
30081 measured reflections	

### Refinement

**Table:**

Refinement on *F*^2^	Primary atom site location: structure-invariant direct methods
Least-squares matrix: full	Secondary atom site location: difference Fourier map
*R*[*F*^2^ > 2σ(*F*^2^)] = 0.054	Hydrogen site location: inferred from neighbouring sites
*wR*(*F*^2^) = 0.204	H-atom parameters constrained
*S* = 0.93	*w* = 1/[σ^2^(*F*_o_^2^) + (0.1425*P*)^2^] where *P* = (*F*_o_^2^ + 2*F*_c_^2^)/3
4624 reflections	(Δ/σ)_max_ < 0.001
187 parameters	Δρ_max_ = 0.68 e Å^−^^3^
55 restraints	Δρ_min_ = −0.51 e Å^−^^3^

### Special details

**Table:**

Experimental. Hydrogen atoms were included in calculated positions with a C—H distance of 0.93 Å and were included in the refinement in a riding motion approximation with *U*_iso_ = 1.2*U*_eq_ of the carrier atom.
Geometry. All e.s.d.'s (except the e.s.d. in the dihedral angle between two l.s. planes) are estimated using the full covariance matrix. The cell e.s.d.'s are taken into account individually in the estimation of e.s.d.'s in distances, angles and torsion angles; correlations between e.s.d.'s in cell parameters are only used when they are defined by crystal symmetry. An approximate (isotropic) treatment of cell e.s.d.'s is used for estimating e.s.d.'s involving l.s. planes.
Refinement. Refinement of *F*^2^ against ALL reflections. The weighted *R*-factor *wR* and goodness of fit *S* are based on *F*^2^, conventional *R*-factors *R* are based on *F*, with *F* set to zero for negative *F*^2^. The threshold expression of *F*^2^ > σ(*F*^2^) is used only for calculating *R*-factors(gt) *etc*. and is not relevant to the choice of reflections for refinement. *R*-factors based on *F*^2^ are statistically about twice as large as those based on *F*, and *R*- factors based on ALL data will be even larger.

### Fractional atomic coordinates and isotropic or equivalent isotropic displacement parameters (Å^2^)

**Table:**

	*x*	*y*	*z*	*U*_iso_*/*U*_eq_	Occ. (<1)
Ag1	1.2500	0.2500	0.5000	0.0834 (2)	
N1	1.11733 (17)	0.16642 (18)	0.44213 (18)	0.0522 (5)	
C1	1.0346 (2)	0.2103 (2)	0.4009 (2)	0.0519 (6)	
H1	1.0308	0.2806	0.4034	0.062*	
C8	0.95133 (18)	0.15564 (19)	0.35281 (18)	0.0456 (5)	
N2	0.95235 (14)	0.05545 (16)	0.34896 (15)	0.0439 (4)	
C7	1.03648 (17)	0.00867 (19)	0.39162 (17)	0.0423 (5)	
C2	1.12040 (17)	0.0612 (2)	0.43877 (18)	0.0457 (5)	
C3	1.2080 (2)	0.0087 (3)	0.4793 (2)	0.0599 (7)	
H3	1.2637	0.0441	0.5099	0.072*	
C4	1.2094 (2)	−0.0938 (3)	0.4727 (3)	0.0680 (8)	
H4	1.2666	−0.1288	0.4990	0.082*	
C5	1.1263 (3)	−0.1472 (2)	0.4270 (3)	0.0661 (8)	
H5	1.1289	−0.2175	0.4234	0.079*	
C6	1.0418 (2)	−0.0988 (2)	0.3877 (2)	0.0527 (6)	
H6	0.9870	−0.1360	0.3581	0.063*	
S1	0.76065 (6)	0.13484 (8)	0.25355 (7)	0.0682 (3)	
C9	0.8619 (2)	0.2074 (2)	0.3038 (2)	0.0526 (6)	
C10	0.8465 (3)	0.3134 (3)	0.2847 (2)	0.0727 (10)	
H10	0.8920	0.3644	0.3053	0.087*	
C11	0.7490 (3)	0.3275 (3)	0.2284 (3)	0.0787 (11)	
H11	0.7233	0.3909	0.2081	0.094*	
C12	0.6976 (3)	0.2410 (3)	0.2073 (3)	0.0757 (11)	
H12	0.6336	0.2394	0.1708	0.091*	
B1	1.0011 (7)	0.5432 (7)	0.4307 (12)	0.174 (6)	0.50
F1	1.0535 (5)	0.4612 (6)	0.4342 (12)	0.234 (7)	0.50
F2	0.9766 (15)	0.5583 (11)	0.5186 (14)	0.405 (13)	0.50
F3	0.9165 (7)	0.5285 (7)	0.3685 (14)	0.331 (10)	0.50
F4	1.0358 (6)	0.6261 (5)	0.4077 (10)	0.244 (7)	0.50

### Atomic displacement parameters (Å^2^)

**Table:**

	*U*^11^	*U*^22^	*U*^33^	*U*^12^	*U*^13^	*U*^23^
Ag1	0.0568 (2)	0.0772 (3)	0.1093 (4)	−0.03177 (17)	0.0040 (2)	−0.01481 (19)
N1	0.0466 (11)	0.0521 (12)	0.0570 (13)	−0.0126 (9)	0.0097 (9)	−0.0046 (9)
C1	0.0569 (15)	0.0423 (12)	0.0570 (15)	−0.0105 (11)	0.0137 (12)	−0.0031 (11)
C8	0.0446 (11)	0.0505 (13)	0.0422 (12)	−0.0028 (10)	0.0109 (9)	−0.0012 (10)
N2	0.0370 (9)	0.0493 (11)	0.0444 (11)	−0.0053 (8)	0.0070 (8)	−0.0035 (8)
C7	0.0390 (10)	0.0458 (12)	0.0432 (12)	−0.0061 (9)	0.0112 (9)	−0.0023 (9)
C2	0.0362 (10)	0.0539 (13)	0.0460 (12)	−0.0082 (9)	0.0073 (9)	0.0011 (10)
C3	0.0417 (13)	0.0758 (19)	0.0595 (16)	−0.0039 (12)	0.0059 (11)	0.0041 (14)
C4	0.0509 (16)	0.081 (2)	0.0702 (19)	0.0120 (15)	0.0093 (14)	0.0139 (16)
C5	0.072 (2)	0.0529 (15)	0.075 (2)	0.0122 (14)	0.0215 (17)	0.0108 (14)
C6	0.0554 (14)	0.0446 (13)	0.0563 (15)	−0.0060 (11)	0.0089 (12)	0.0045 (11)
S1	0.0499 (4)	0.0782 (6)	0.0718 (5)	0.0075 (3)	0.0036 (3)	0.0082 (4)
C9	0.0533 (14)	0.0571 (15)	0.0479 (14)	0.0086 (12)	0.0124 (11)	−0.0011 (12)
C10	0.079 (2)	0.082 (2)	0.0557 (17)	0.0280 (19)	0.0122 (16)	−0.0003 (15)
C11	0.085 (2)	0.076 (2)	0.075 (2)	0.032 (2)	0.0171 (19)	0.0093 (18)
C12	0.066 (2)	0.087 (3)	0.071 (2)	0.0307 (18)	0.0072 (17)	0.0093 (16)
B1	0.057 (5)	0.087 (6)	0.361 (17)	−0.015 (4)	0.015 (8)	−0.096 (9)
F1	0.063 (4)	0.091 (5)	0.53 (2)	−0.003 (3)	0.017 (7)	−0.064 (8)
F2	0.66 (4)	0.145 (10)	0.48 (2)	−0.088 (16)	0.29 (2)	−0.150 (14)
F3	0.130 (7)	0.121 (7)	0.64 (3)	−0.002 (5)	−0.133 (11)	−0.059 (11)
F4	0.125 (6)	0.077 (4)	0.55 (2)	−0.037 (4)	0.123 (10)	−0.048 (7)

### Geometric parameters (Å, º)

**Table:**

Ag1—N1	2.166 (2)	C5—C6	1.355 (4)
Ag1—N1^i^	2.166 (2)	C5—H5	0.9300
N1—C1	1.313 (4)	C6—H6	0.9300
N1—C2	1.391 (3)	S1—C12	1.705 (3)
C1—C8	1.411 (4)	S1—C9	1.731 (3)
C1—H1	0.9300	C9—C10	1.432 (5)
C8—N2	1.323 (3)	C10—C11	1.432 (6)
C8—C9	1.460 (4)	C10—H10	0.9300
N2—C7	1.350 (3)	C11—C12	1.349 (6)
C7—C2	1.400 (3)	C11—H11	0.9300
C7—C6	1.421 (4)	C12—H12	0.9300
C2—C3	1.418 (4)	B1—F4	1.267 (11)
C3—C4	1.357 (5)	B1—F1	1.307 (11)
C3—H3	0.9300	B1—F3	1.317 (11)
C4—C5	1.391 (5)	B1—F2	1.343 (13)
C4—H4	0.9300		
			
N1—Ag1—N1^i^	179.998 (2)	C6—C5—H5	119.4
C1—N1—C2	117.2 (2)	C4—C5—H5	119.4
C1—N1—Ag1	123.11 (18)	C5—C6—C7	120.3 (3)
C2—N1—Ag1	119.34 (18)	C5—C6—H6	119.8
N1—C1—C8	122.9 (3)	C7—C6—H6	119.8
N1—C1—H1	118.5	C12—S1—C9	90.6 (2)
C8—C1—H1	118.5	C10—C9—C8	128.5 (3)
N2—C8—C1	120.9 (2)	C10—C9—S1	112.9 (2)
N2—C8—C9	117.7 (2)	C8—C9—S1	118.5 (2)
C1—C8—C9	121.4 (2)	C9—C10—C11	108.3 (4)
C8—N2—C7	117.1 (2)	C9—C10—H10	125.8
N2—C7—C2	123.1 (2)	C11—C10—H10	125.8
N2—C7—C6	119.3 (2)	C12—C11—C10	114.4 (4)
C2—C7—C6	117.7 (2)	C12—C11—H11	122.8
N1—C2—C7	118.7 (2)	C10—C11—H11	122.8
N1—C2—C3	120.3 (2)	C11—C12—S1	113.8 (3)
C7—C2—C3	120.9 (3)	C11—C12—H12	123.1
C4—C3—C2	119.0 (3)	S1—C12—H12	123.1
C4—C3—H3	120.5	F4—B1—F1	118.4 (9)
C2—C3—H3	120.5	F4—B1—F3	108.1 (11)
C3—C4—C5	120.8 (3)	F1—B1—F3	109.0 (9)
C3—C4—H4	119.6	F4—B1—F2	106.8 (10)
C5—C4—H4	119.6	F1—B1—F2	110.4 (12)
C6—C5—C4	121.3 (3)	F3—B1—F2	103.0 (10)

Symmetry code: (i) −*x*+5/2, −*y*+1/2, −*z*+1.
